# Complex DNA Damage: A Route to Radiation-Induced Genomic Instability and Carcinogenesis

**DOI:** 10.3390/cancers9070091

**Published:** 2017-07-18

**Authors:** Ifigeneia V. Mavragani, Zacharenia Nikitaki, Maria P. Souli, Asef Aziz, Somaira Nowsheen, Khaled Aziz, Emmy Rogakou, Alexandros G. Georgakilas

**Affiliations:** 1 DNA Damage Laboratory, Physics Department, School of Applied Mathematical and Physical Sciences, National Technical University of Athens, Zografou Campus, 15780 Athens, Greece; ifimav@mail.ntua.gr (I.V.M.); znikitaki@mail.ntua.gr (Z.N.); mariasouli@mail.ntua.gr (M.P.S.); 2 Department of Pediatrics and Adolescent Medicine, Mayo Clinic, Rochester, MN 55905, USA; asefaziz@gmail.com; 3 Mayo Medical Scientist Training Program, Mayo Medical School and Mayo Graduate School, Mayo Clinic, Rochester, MN 55905, USA; somairanowsheen@gmail.com (S.N.); aziz.khaled19@gmail.com (K.A.); 4 Department of Molecular Pharmacology and Experimental Therapeutics, Mayo Clinic, Rochester, MN 55905, USA; 5 First Department of Pediatrics, “Aghia Sophia” Children’s Hospital, Medical School, University of Athens, 11527 Athens, Greece; emmyrogakou@gmail.com

**Keywords:** ionizing radiation effects, DNA damage and repair, complex DNA damage, carcinogenesis, immune response, radiation therapy

## Abstract

Cellular effects of ionizing radiation (IR) are of great variety and level, but they are mainly damaging since radiation can perturb all important components of the cell, from the membrane to the nucleus, due to alteration of different biological molecules ranging from lipids to proteins or DNA. Regarding DNA damage, which is the main focus of this review, as well as its repair, all current knowledge indicates that IR-induced DNA damage is always more complex than the corresponding endogenous damage resulting from endogenous oxidative stress. Specifically, it is expected that IR will create clusters of damage comprised of a diversity of DNA lesions like double strand breaks (DSBs), single strand breaks (SSBs) and base lesions within a short DNA region of up to 15–20 bp. Recent data from our groups and others support two main notions, that these damaged clusters are: (1) repair resistant, increasing genomic instability (GI) and malignant transformation and (2) can be considered as persistent “danger” signals promoting chronic inflammation and immune response, causing detrimental effects to the organism (like radiation toxicity). Last but not least, the paradigm shift for the role of radiation-induced systemic effects is also incorporated in this picture of IR-effects and consequences of complex DNA damage induction and its erroneous repair.

## 1. Introduction

Many decades of experimental research in cellular and molecular radiation biology have provided evidence suggesting that DNA damage plays a critical role in a plethora of human pathologies, including cancer, premature aging and chronic inflammatory conditions [1]. In response to both endogenous and exogenous insults (approximately 10^4^–10^5^ lesions induced per cell per day) mammalian cells evolve the DNA damage response and repair pathway (DDR/R) that arouse the immune system, activating DNA damage checkpoints and facilitating the removal of DNA lesions [1]. Dysregulation of the DDR/R pathway is closely linked to several human disorders associated with cancer susceptibility, developmental abnormalities, neurodegenerative disorders and accelerated aging [2,3,4].

The DDR is triggered by a wide variety of physico-chemical aberrations in the genome. Depending on the source of damage, diverse lesions in the DNA can be induced, including nucleotide alterations (mutation, substitution, deletion and insertion), bulky adducts, single strand breaks (SSBs) and double strand breaks (DSBs) [5]. Genotoxic agents, such as ultraviolet light from the Sun and IR from e.g., cosmic radiation and medical treatments utilizing X-rays or γ-radiation, mainly cause changes or losses of bases (abasic sites), crosslinks formed between two complementary DNA strands, SSBs and DSBs. Such types of DNA damage can occur separately or in conjunction with one another, resulting in complex DNA damage (clustered lesions). Chemical agents used in cancer therapy can also induce a diversity of DNA lesions, such as intrastrand or interstrand crosslinks[6]. Apart from these environmental agents and genotoxic chemicals, DNA aberrations can also arise from physiological processes such as base mismatches introduced during DNA replication [7] and from the release of reactive oxygen and nitrogen species (ROS/RNS) upon oxidative respiration or through redox-cycling events mediated by heavy metals [8]. Additionally, replication stress resulting from oncogenic signaling may cause genome instability [9].

It is well-accepted that IR can induce cancer even at clinically relevant doses and the relationship between radiation and formation of solid tumors is considered to be linear in the dose range of 0.15–1.5 Gy [10]. Epidemiological data from the Life Span Study of the Japanese Atomic Bomb survivor cohort has provided significant evidence on the causal relationship between IR exposure and carcinogenesis [11,12]. For low doses (<0.1 Gy), there is a heated debate on the actual relationship between dose and cancer incidence. Even recently the validity of the well-known linear no-threshold (LNT) model has been challenged and many questions are still open regarding if the radiosensitivity of a tissue to malignant transformation increases or decreases with dose and if the actual form of the curve, i.e., linear or curvilinear, etc. [13,14].

Our knowledge of the mechanistic basis of the strong link between IR and carcinogenesis has been based on early studies using various animal models and it is concluded that radiation tumorigenesis proceeds in a conventional multi-step mode following radiation-induced key gene losses from single-target cells (including possible stem cells) [15]. These genes can be DNA damage response, apoptotic and cell cycle control genes and others. This radiation-induced GI can be transmitted over many generations after irradiation via the progeny of surviving cells [16]. Complex DNA damage and the consequent less precise and/or delayed DNA repair certainly hold a pivotal role(s) in this association between IR and cancer [17,18]. Last but not least, in order to draw the current picture of the factors contributing to radiation-induced carcinogenesis one should also add the non-targeted effects and the release of clastogenic factors in non-irradiated cells and tissues [19,20], as well as the involvement of inflammation and constant triggering of the immune system [21,22].

Lesions formed in a close proximity (i.e., within a few nm) result in clustered types of DNA damage, also called multiply damaged sites (MDS) and are considered the fingerprint of IR. Clustered DNA lesions can comprise a DSB and several base damages and/or abasic sites in close vicinity. In the case of multiple DSBs, we refer to the idea of complex DSBs [23]. The biological significance of such lesions relates to the inability of cells to process them efficiently compared to isolated DNA damages and the outcome in case of erroneous repair can vary from mutations up to chromosomal instability [24,25,26]. Therefore one should wonder if there are any mutational signatures of IR. Only recent evidence, mostly due to availability and affordability of next generation sequencing technologies, indicates that such radiation signatures do exist [27]; yet previous studies have shown the lack of such associations [28]. Specifically, Behjati et al. have shown a significant increase in small chromosome deletions and balanced inversions in radiation-associated tumors which probably act as driver mutations and explain the carcinogenic potential of IR. More importantly, they suggest that these chromosomal abnormalities originate from the repair of radiation-induced DNA damage via the less accurate pathways of non-homologous (NHEJ) or microhomology mediated end-joining (MMEJ) [27]. Therefore, accepting the claim that IR induces complex DNA damage that is irreparable and leads to mutations or structural abnormalities and subsequently to genomic instability (GI) and cancer, radiation-induced cancers should bear traces of the radiation-related origin of these mutations. Disruption of genome maintenance (i.e., GI) can occur through a variety of mechanisms and it is now considered as a key hallmark of cancer. Hence, there is a great need for improved detection techniques at cellular and tissue level that will provide valuable information for understanding the cellular mechanisms to process clustered DNA lesions [29].

## 2. Clustered DNA Lesions. A Challenge to Detect, a Challenge to Repair

### 2.1. Biological Significance and Detection of Clustered DNA Damage

The complexity of DNA damage as discussed above refers to the idea of clustering of several and different DNA lesions within a short DNA region of 10–15 bp. The two main categories of lesions appearing in a cluster are the DSB and non-DSB lesions, usually referred to as *oxidatively-clustered DNA lesions* (OCDLs). The reader can refer to several comprehensive reviews for the general description of clustered DNA damage [23,26,30], detection methodologies [23,29,31] and biological importance. More specifically for the accepted repair resistance of these lesions, including experimental evidence on the increase of the possibility for generation of mutations and chromosomal breaks after erroneous repair of clustered DNA lesions please see [17,25,26,32,33]. Although within the context of this review we refer to bistranded DNA lesions appearing in both the DNA strands, there is also the possibility of unistranded or tandem lesions appearing in the same DNA strand and several groups have dealt with the processing and biological role of these complex DNA lesions as described in recent reviews [31,34,35,36].

The biological significance of clustered DNA damage is not only based on the difficulty encountered by the different DNA repair proteins that process these closely spaced DNA lesions, but also to the fact that several of these OCDLs can be converted into de novo DSBs during repair [37,38]. There are still many questions open as to which type of clusters will be more prone to be converted to potentially dangerous DSBs, but some parameters such as the presence of an SSB in one strand that delays the simultaneous process of other base lesions on the other strand, the nucleotide distance between the various lesions and the direction 3′ or 5′ to each other have been found to be critical [25,39].For example, some recent in vitro data using plasmid pUC18 DNA exposed to high-LET IR (He^2+^ or C^6+^ ions) or low-LET (X-rays) and under varying radical-scavenging conditions, suggest that base lesion clusters appear three or more base pairs apart and are promptly converted to a DSB by a glycosylase, regardless of the order of enzymatic treatment [40]. These and other similar results are in good agreement with Monte Carlo (MC) track structure calculations, suggesting an increase of complexity with LET and specific base to SSB ratio etc. [41]. Additionally, one cannot disregard that the initial repair steps at clustered damage sites is a major parameter that directs towards the conversion of MDS into DSB or not [42]. Unrepaired clustered DNA lesions can lead to chromosomal breaks and significant GI as primarily manifested during the induction of clustered DNA damage by high-LET radiations [43].

The experimental validation of DNA damage clustering induction, as well as, the repair mechanisms involved have not been an easy task. There are some discrepancies that can still be found between experimental evidence or data and prediction models using Monte Carlo (MC)-based methodologies [41,44,45]. Significant advancement in the understanding of expected clustered DNA damage induction mechanisms has been achieved using a fast and cell-level MC code, the Monte Carlo Damage Simulation (MCDS) code, integrated into the general-purpose MC N-particle radiation transport code system (MCNP) [46]. At the same time, a better understanding of the processes and mechanisms involved in the repair of clustered DNA lesions has been provided by the development of analytical biochemical models for DSB and base lesion repair [47,48,49].

Towards the history and advances in the field of experimental detection of clustered DNA lesions, the reader can refer to the above mentioned references. Current research in this field is based on the idea that theory and predictions do not always coincide with experimental evidence. The major challenges towards the detection of clustered DNA damages have been: (1) the accurate measurement of DSBs and OCDLs levels and their types, especially at the cellular level and (2) theirin situ detection and reliable quantitative measurement.

**Figure 1:**
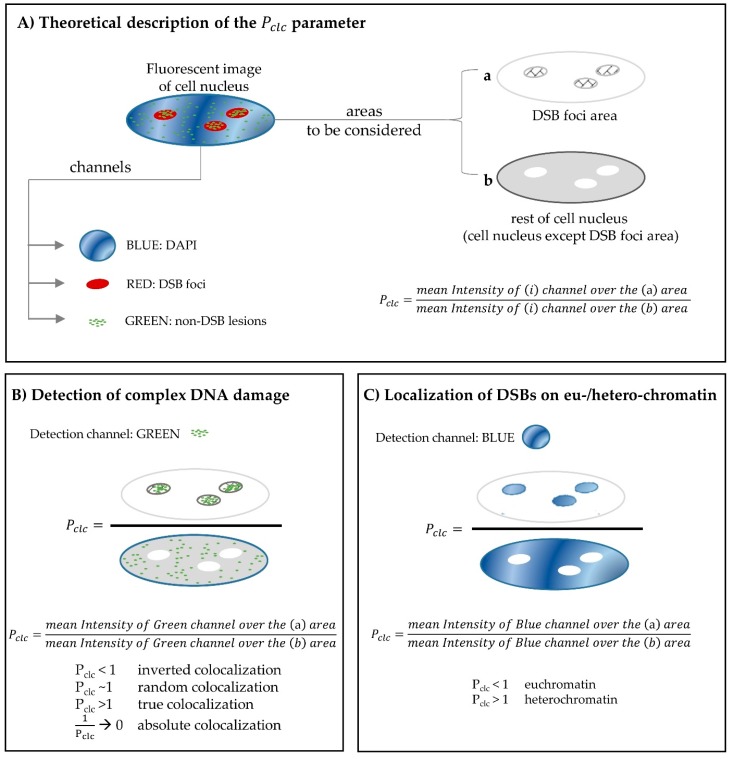
Optimization of fluorescence microscopy and colocalization assays for detection of complex DNA damage. (**A**) Theoretical description of the *Pclc* parameter [64]: An RGB fluorescent image can be split into three channels (Blue: DAPI, Red:DSB foci and Green: non-DSB lesions), and can also be considered as consisting of two geometrical areas: (a) the DSB foci area and (b) the rest of cell nucleus, i.e., the cell nucleus area after the exclusion of DSB foci area. The colocalization parameter *Pclc* examines the mean Intensity of a given fluorescent channel (i) considered over the DSB foci area, with its mean Intensity considered over the rest of the cell nucleus. (**B**) Detection of complex DNA damage: Complex DNA damage can be detected by considering the mean Intensity of the channel that corresponds to non-DSB lesions (Green channel here) for the calculation of *Pclc* parameter. Any *Pclc* values significantly greater than 1 imply true colocalization and subsequently enable the detection of complex DNA lesions. (**C**) An additional suggested application of *Pclc* parameter-localization of DSBs on eu-/hetero-chromatin: Mean intensity of DAPI channel (blue) over the DSBs area divided by the mean intensity of the same channel over the rest of cell nucleus (after excluding any nucleoli areas). *Pclc* values less than 1 imply DSB foci localization on euchromatin DNA regions, where the DAPI intensity is expected to be lower. In each case, measurement of lesions is being performed indirectly by the use of DNA damage/repair proteins specific primary antibodies (e.g., against γ-H2AX:DSB or OGG1:oxidized purines etc.) detected by the appropriate fluorescent labelled secondary antibodies as described in the text.

**Figure 2:**
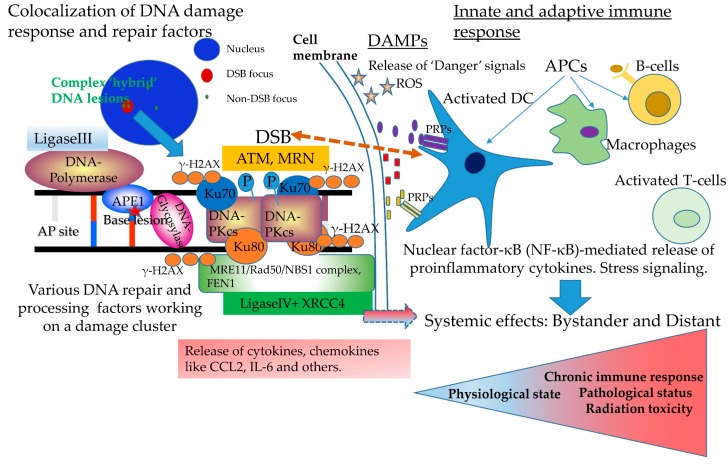
Linking processing of clustered DNA damage and immune response. I. The challenge of repairing a clustered damaged DNA site: a task for real survivors. Upon the induction of clustered DNA damage consisting for example of one double strand break (DSB) and two oxidative DNA lesions like a damaged base (shown here with an asterisk) and an apurinic/apyrimidinic (AP) site, two at least DNA repair pathways and several DNA repair proteins will arrive at the same chromosome region. For the base damage, the primary pathway is the base excision repair (BER) while for the DSB, here we consider for simplicity only the non-homologous end joining (NHEJ). In all cases, the most basic proteins and enzymes are also described in the main text. Last but not least, as shown by advanced fluorescence microscopy and foci colocalization, each DSB is expected to be rapidly accompanied by the phosphorylation of thousands of H2AX histone protein molecules called γH2AX. The MRN complex functions rather as a sensor of DNA ends and activates ATM kinase. The ATM phosphorylates substrates such as Chk2, p53, and the H2AX in flanking chromosomal regions. II. Linkage to immune response. Processing of clustered DNA damage and especially of unrepaired orpersistent is expected to lead to senescence or cell death i.e., apoptosis, necrosis (accidental, non-programmed), and necroptosis (programmed). All these processes can trigger the extracellular release of diverse signatures of ‘Danger’ signals or Damage-Associated Molecular Patterns (DAMPs: ATP, short DNAs/RNAs, ROS, heat shock proteins (HSPs), high-mobility group box 1 (HMGB)-1, S100 proteins and others) [65]. DAMPs activate different pattern recognition receptors (PRPs) including for example Toll-like receptors (TLRs) and inflammasomes, a process that leads usually to inflammation and immune-related pathologies. Interestingly, recent evidence as explained in the main text, suggests a direct interactions between different PRPs and DNA repair proteins involved in DSB repair and others (Dashed arrow connecting DSB to PRPs). Cellular damage or death can also lead to the release of several cytokines and chemokines that can regulate immune responses. Activation of PRPs usually results in nuclear factor-κB (NF-κB)-mediated release of various proinflammatory cytokines like IL-6, IL-8 and others. The activation of antigen-presenting cells (APCs) like dendritic cells, macrophages will induce primarily the innate immune response (activation of T-cells) and most rarely by B-cells, the adaptive immune response. In all cases, the constituent and constant triggering of the immune system is expected to generate a variety of systemic effects on the organism and possibly pathophysiology, close to the damaged cells often called as “bystander” effects or distant. Overall, for the final assessment of radiation effects and the return to the physiological state, the role of immune response and the systemic nature of radiation is of enormous importance.

During the last decade and since its initial discovery in 1998, the application of the γH2AX methodology has provided a significant boost towards reliable measurements of DSBs at a cellular or tissue level [50,51,52,53,54,55,56,57]. On the other hand, for the measurement of non-DSB lesions at least in situ, significant advancements have been made using adaptations of fluorescence microscopy and foci colocalization as reviewed in [29], but still there is no reliable in situ-technique to detect closely spaced DNA lesions within 1–20 bp apart. The colocalization of two or more antibodies (corresponding to DNA repair proteins presumably working on a clustered damage site), certainly provides valuable information, but this only gives an idea on how many different proteins maybe present in a chromosome region of a few Mbp. In each case, measurement of DNA lesions is being performed indirectly by the use of usually two DNA damage/repair proteins specific primary antibodies (e.g., against γ-H2AX:DSB and OGG1:oxidized purines or NTH1:oxidized pyrimidines etc.) each detected by the appropriate fluorescent labeled secondary antibodies. The simultaneous use of more than three different antibodies requires highly advanced microscopic systems and it is considered to be highly challenging. This microscopy-based methodology however, is very distant from the original definition of clustered DNA damage located in a very small DNA region [29]. Based on the above and in an attempt to make a rough comparison between the originally used adaptations of gel electrophoresis to measure different types of DNA clusters (DSBs and non-DSBs) as introduced by Sutherland and colleagues [58,59,60] and afterwards by others [38,61,62] one can conclude that: (1) there are two main methodologies to measure complex DNA lesions at the cellular level; one based on DNA fragmentation measurement using gel electrophoresis with repair enzymes as damage probes, and in situ immunofluorescence microscopic approaches using different antibodies to allow foci colocalization centered around the DSB focus (usually γH2AX/53BP1) (Figure 1 and Figure 2) both methodologies are necessary and useful, but they are complementary; when it comes to measurement of damage complexity of DSBs and non-DSBs one should consider applying them both. A short description on the powerful γH2AX methodology follows.

### 2.2. The Epigenetic Biomarker γH2AX Detects DNA Double-Strand Breaks

To date, a large volume of studies supports the notion that the γH2AX epigenetic biomarker has been established as the most sensitive and specific epigenetic biomarker for DSB detection and quantification. H2AX is a mammalian variant that belongs to the H2A histone family that has a phosphorylation site at a serine 139. This site becomes rapidly phosphorylated when DSBs are generated into DNA. It has been well documented that this phosphorylation is specific to DSBs [57]. This specific phosphorylation is denoted as “γ-phosphorylation” and the H2AX histone molecules that “carry” this phosphorylation are designated as “γH2AX” accordingly.

One of the most intrinsic features of γH2AX is that γ-phosphorylation extents at megabase-long domains in chromatin. The γ-phosphorylation of H2AX is evident within minutes after the generation of DSBs. Nevertheless, γ-phosphorylation is not restricted to the vicinity of the sites of the DSB, but extends both sides of the damage, and reaches megabase-long domains in chromatin [53,63]. This feature of γ-phosphorylation is very important; it represents a biological amplification mechanism where one DSB induces the γ-phosphorylation of thousands of H2AX molecules along megabase-long domains of chromatin that are adjusted to the sites of DSBs. The γ-phosphorylated megabase-long chromatin domains that are adjusted to the sites of one DSB are the basis for a very important technological implication. As one DSB is surrounded by thousands of γ-phosphorylated H2AX nucleosomes, specific antibodies enable the microscopy observation of the site of one DSB by immunocytochemistry. When detected with epifluorescence or confocal microscopy, γH2AX foci appear as large, roughly spherical conformations in cells that are in the G0, G1, S, or G2 phase of the cell cycle [63].

On the contrary, it has been demonstrated that γH2AX foci appear as band-like conformations [63] in deer mitotic cells, resembling perhaps the known bands in human mitotic chromosomes as seen in routine karyotype tests. Though, these conformations have not been detected in human mitotic cells, perhaps due to intrinsic features of human mitotic chromatin. Additionally, the possible detection of only one DSB in the nucleus by γH2AX immunocytochemistry [66] renders this technology as currently the most sensitive assay for the detection of DSBs.

Although the amount of H2AX, as well as the percentage of H2AX in respect to the total H2A of the histone family is not the same between differentiated cell types, the percentage of chromatin that becomes phosphorylated per one DSB has a roughly constant average. That permits the quantification of the γH2AX foci [63] in cell lines, primary cells, and tissues. Assays based on specific antibodies against the characteristic γH2AX epitope (e.g., confocal and epifluorescent microscopy, flow cytometry, ELISA, immunoprecipitation etc.) have been incomparably successful for the detection of DSBs [51,67,68,69]. Among them, immunocytochemical detection of γH2AX has become the primary method of detection, as it is several orders of magnitude more sensitive than other methods and has the potential for quantification [56]. In addition, it has been shown that γH2AX foci are formed preferentially in euchromatin after IR-exposure [70].

In general, the γH2AX assays share four important technical features: (i) a general acceptance for specificity to DSBs, (ii) sensitivity, (iii) quantification of DSBs, and (iv) repeatability and reproducibility. Regarding the technical supremacy of the specific methodology one can assign the following features: (i) specificity to DSBs: the γH2AX has been shown to detect specifically DSBs rather than other DNA damages [57]. However, it has been reported that γH2AX can be formed at other types of lesions and in high frequencies in S-phase cells undergoing replication [71], or some other cell types undergoing for example chromatin remodeling [72], (ii) sensitivity: immunoassays utilizing specific antibodies for γH2AX show the highest score in sensitivity. Even one DSB can be detected by anti-γH2AX immunocytochemistry [66]. The biology of γ-phosphorylation provides the explanation for this remarkable sensitivity; visualization of only one DSB in the whole nucleus is feasible, as γ-phosphorylation spans megabaselong domains in chromatin juxtaposed to the break, (iii) quantification: the presence of γH2AX detected by antibody based techniques can be quantified by various methods, such as confocal and epifluorescence microscopy (measured manually or automatically), flow cytometry, western blot quantification, etc. [51,67] and (iv) repeatability and reproducibility: to date, the repeatability and reproducibility of the method have been demonstrated by numerous diverse research laboratories all over the world, as demonstrated by the number of scientific publications [50,73,74,75,76,77,78,79,80,81,82].

At this point, it must be mentioned that a variety of tumor cells have been found with increased numbers of γH2AX foci suggesting to be related to the overall chromosomal instability of these cells [83]. Last but not least, it has been also indicated by Banath et al. that persistence of DNA damage-induced γH2AX foci can be suggestive of lethal DNA damage so that it may be possible to predict tumor cell killing by different DNA damaging therapeutic agents by measuring the fraction of cells that retain γH2AX signalling [84].

### 2.3. Using Fluorescence Microscopy for the in situ Detection of Complex DNA Damage. A Useful Tool

The study of complex DNA damage in terms of in situ detection involves the concept of DNA repair colocalization (DNA repair centers) as previously introduced for DSBs [43,85] and non-DSB damage [29,43,64]. The term “colocalization” actually refers to the spatiotemporal coexistence of two or more proteins of different type. The detection of complex DNA lesions consisting of a variety of DSBs and OCDLs is made possible through the visualization of proteins participating in a distinct DNA repair mechanism, e.g. one protein participating in the base excision repair (BER) processing base lesions and another one participating in the homologous recombination (HR) or the non-homologous end joining (NHEJ) for the repair of DSBs. As shown in Figure 2, upon the induction of a cluster of DNA lesions, several DNA repair pathways and proteins will be involved. For short-patch BER, a DNA glycosylase will arrive, excise the damaged base and the repair will be completed presumably by the human AP endonuclease 1 (APE1), a polymerase and ligase III to seal the broken ends. In the nearby DSB area (within a few bp apart), the Ku heterodimer (Ku70/80) initiates NHEJ by binding to the free DNA ends and engaging other NHEJ factors such as DNA-dependent protein kinase (DNA-PK), XRCC4, and DNA Ligase IV to the site of the break. DNA-PK becomes activated upon DNA binding, and phosphorylates a number of substrates including p53, Ku, and DNA Ligase IV cofactor XRCC4. Phosphorylation of these factors is believed to further facilitate the processing of the break. Finally, in order for ligation to occur, a partial processing of the ends by nucleases Artemis, MRE11/Rad50/NBS1 complex and FEN-1 is taking place.

Although the in situ immunofluorescence has been extensively utilized for the detection of single/simple DNA damage including one type of lesions [74,86,87,88], the simultaneous detection of DSBs and non-DSB lesions has been reported only in a few studies [43,64,89]. The difficulty in achieving visualization of base lesions, in terms of foci, lies in the fact that only a few molecules of every specific DNA repair protein (e.g., OGG1, NTH1, APE1 etc.) are taking part in the repair of a single lesion, in contrast with DSB repair where hundreds/thousands of molecules of the same DNA repair protein (like γH2AX/53BP1) may contribute to the process, as discussed above. Moreover, unlike γH2AX protein which becomes present mainly upon a DSB formation, most of the non-DSB repair proteins have endogenous concentrations within the cell nucleus, therefore resulting in increased background signal. A pre-extraction step in the experimental procedure, as well as the introduction of the *Pclc* colocalization parameter in image analysis [64] have helped researchers overcome these obstacles (Figure 1). In Figure 1, the theoretical description of the *Pclc*-parameter is given in detail (panel A), along with its application for the detection of complex DNA damage (panel B) and an additional application for the derivation of useful data regarding the localization of DNA repair proteins in euchromatin/heterochromatin regions (panel C).

## 3. Complex DNA Damage, Immune Signaling and Systemic Effects. A Puzzling Case of Triage for the Cell

Triage in medical situations refers to the assignment of degrees of urgency to wounds or illnesses to decide the order of treatment of a large number of patients or casualties. Radiation injury for the cell can be considered as a major “wound to its crucial organs” and in many cases a matter of life or death. The delineation of how DDR exerts immune responses still can be considered as a puzzling topic [90]. Based on the above ideas, it is generally accepted that once complex and/or persistent DNA damage is induced and most probably GI, immune signaling is initiated by different components of the DDR/R pathway including DNA damage sensors, transducer kinases, effectors and repair proteins [3]. In general, association between innate immune system response and persistent DNA damage has been shown in various cases as reviewed in [91,92]. In the same direction, Ermolaeva et al. used the nematode *Caenorhabditis elegans* eukaryotic system to show that DNA damage in germ cells induces an innate immune response that consequently leads to activation of the ubiquitin-proteasome system (UPS) in somatic tissues, which confers enhanced proteostasis and systemic stress resistance [93]. Rodier et al. have shown that X-ray damaged human HCA2 fibroblasts develop persistent chromatin lesions bearing DSBs detected using γH2AX/53BP1 foci as surrogate markers, which triggers the secretion of inflammatory cytokines such as interleukin-6 (IL-6) [94]. It is important to notice, that this cytokine secretion occurred only after establishment of persistent and heavy DNA damage (10 Gy of X-rays), associated with senescence and not after transient DNA damage responses (X-ray dose of 0.5 Gy). On the other hand, systemic DNA damage responses are part of the organism’s defense system in order to secure removal of damaged and malfunctioning cells and preserve tissue integrity and functionality i.e., tissue homeostasis [95]. For example, it has been shown that in repair deficient Ataxia-telangiectasia (AT) patients, where the repair protein ATM is defective, small DNA fragments generated from the excessive DNA-breaks accumulate in the cytoplasm of these patients' cells. The DNA fragments are consequently recognized by innate immune receptors that normally detect viral DNA. This “false alarm” of viral invasion results in the production of type I interferon which in turn drives the innate immune system into an activated state [92].

Regarding IR exposure as a genuine genotoxic stress, accumulating experimental evidence suggests a diverse range of radiation effects for non-irradiated areas often referred to as non-targeted effects (NTE) or under the general umbrella of systemic effects [65,96,97,98]. The NTE can be separated in two major groups: near (bystander), where non-irradiated cells exhibit a response similar to their neighboring irradiated cells, and distant (e.g., the clinically relevant abscopal effect) while different mechanisms are implicated in each case, as discussed recently in [65,96]. The NTE usually involve the discharge of various chemical and biological mediators from the irradiated cells and thus promoting the communication of the radiation attack via the so-called damage-associated molecular patterns (DAMPs), which is based on the originally introduced idea of “danger” signals [99].

Recent work by Redon et al. showed that growing tumors may act as a type of stress in the organism and induce complex DNA damage (DSBs and OCDLs) in distant proliferative tissues in vivo [100]. According to this study, rapidly growing normal tissues, such as colon and skin were found to be particularly susceptible to remotely induced DNA damage and a signaling molecule involved in inflammation, the chemokine CCL2 (monocyte chemoattractant protein-1: MCP-1) appeared to be a major player in promoting this distant effect. Interestingly, later studies by the same groups showed that this systemic DNA damage accumulation under tumor growth can be inhibited by the antioxidant Tempol suggesting the involvement of oxidative stress [101]. The involvement of CCL2 and macrophage activation in tumor-induced distant DNA damage suggests some resemblances with the chronic tissue stress responses usually referred to as para-inflammation [102], which relies mostly on alternatively activated macrophages (M2) rather than on classically activated macrophages (M1) associated with the acute inflammatory response [103].

A CCL2-based mechanism has been also suggested for other cases of stresses i.e., exposure to IR. Specifically, it was shown that a single-dose whole-body γ-irradiation (8 Gy) induced DNA damage in mice neuronal retina, which was complemented by a low-grade chronic inflammation, para-inflammation, characterized by upregulated expression of chemokines (CCL2, CXCL12, and CX3CL1) and microglial activation [104]. Recent patient studies also suggest an actual involvement of cytokines in the induction of RT-induced systemic DNA damage in normal tissues distant to the irradiation site [105]. More specifically in this study, sixteen patients with non-small cell lung carcinoma (NSCLC) received 60 Gy in 30 fractions of definitive thoracic RT with or without concurrent chemotherapy (chemoRT) and peripheral blood lymphocytes (PBL) and eyebrow hairs samples were taken prior, during, and after RT. The results showed an elevation of DSBs manifested as γH2AX foci in PBL, representing normal tissues in the irradiated thorax volume, 1 hour after fraction one and γH2AX foci numbers returned to near baseline values in 24 hours after treatment. Most importantly, unirradiated hair follicles, exhibited delayed systemic (abscopal) DDR measured as γH2AX foci which increased at 24 hours post-fraction one, and remained elevated during treatment in a dose-independent manner. This distant radiation effect was related with changes in plasma levels of MDC/CCL22 and MIP-1α/CCL3 cytokines. Interestingly and consistent with the unifying model suggestion introduced by Georgakilas uniting different types of stress i.e., radiations and a growing tumor [106], MCP-1 blockade by neutralizing antibodies was found to inhibit lung cancer tumor growth by altering macrophage phenotype and activating cytotoxic CD8^+^ T lymphocytes (CTLs) [107]. Another side of the same coin of cytokines-inflammation is the reverse activity. Earlier studies have shown that ROS/RNS could be generated in vitro by a mixture of inflammatory cytokines (IL-1β, IFN-γ and tumor necrosis factor α) in three human cholangiocarcinoma cell lines by a nitric oxide (NO)-dependent response, as assessed by alkaline (denaturing) comet assay [108]. In addition, a parallel inhibition of global DNA repair activity by 70% was detected. These and later data indicate that activation of iNOS and excess production of NO in response to inflammatory cytokines can cause DNA damage and inhibit DNA repair, at least partially. Recent extensive bioinformatics-based metanalysis studies have verified the interactions between mediators of systemic effects and DDR/R components, as well as interactions between pattern recognition receptors (PRPs) and DNA repair proteins like BRCA1, XRCC1, DNA-PK, Ku70/80 and others [96,109]. Recently, Nikitaki et al. produced a detailed list of proteins implicated in different categories of radiation-induced systemic effects, including the clinically relevant abscopal phenomenon, using improved text-mining and bioinformatics tools from the literature. Genes belonging to the DDR/R pathway and protein-protein interaction (PPi) networks as well as KEGG pathway analyses have revealed that the main pathways participating in NTE are: apoptosis, TLR-like and NOD-like receptor signaling pathways [96].

Conclusively, one can wonder how cells triage this scenario of the interaction between complex DNA damage, immune signaling and systemic effects, which is the most important in regulating the overall outcome of this complex crosstalk (Figure 2). It is rather secure to suggest that complex and persistent DNA damage constitutes a major “danger” signal for the cells and this probably alarms the whole cell or tissue about something “peculiar” happening in this area of damage. If this complex form of damage is processed correctly and all problems have been resolved then the alarm goes off, but the “danger” signaling may already have generated an immune response. In this case, the outcome is uncertain. Immune response manifested initially at least as innate and later on as adaptive and inflammation maybe present, especially when specific “danger” signals are produced due to cell death or senescence. As so, a continuing systemic effect of unknown severity and duration will be induced resulting to a chronic state of immune response and a precursor of pathological evolution and disease as presented with red in Figure 2. Recent evidence obtained using mice carrying an ERCC1-XPF DNA repair defect systematically or in adipocytes, suggests that persistent DNA damage-driven autoinflammation plays a causative role in adipose tissue degeneration, with important complications for advanced lipodystrophies and aging [110]. In any case, the knowledge of the exact mechanisms and mediators of systemic responses will be very useful in various applications that involve complex DNA damage formation, such as RT, chemotherapy and tumor growth early detection. As nicely presented in a recent work by Pateras et al. continuous triggering of DDR/R can lead to excessive innate and adaptive immune response which, in turn, can lead to pathological conditions and disease [109].

## 4. Clinical Implications of Complex DNA Damage

As well-known, IR exposure can be considered for humans as a double-edged sword to either hurt or save. On one hand it can induce significant levels of complex and usually unrepairable DNA damage that can lead to enhanced mutation levels, GI and cancer, but on the other hand it can be used as the ultimate weapon against tumors [39]. Treatment options for patients with various kinds of malignancies have expanded with discoveries of druggable targets as well as technological advances. Surgical resection, chemotherapy and RT are the three major available modalities for the treatment of most cancers and are utilized either in combination or separately, as deemed appropriate. In case of chemotherapy and RT, the main aim is to spare normal cells while inducing sufficient, non-repairable DNA damage in tumor cells. Consequently, cancer cells may exit the cell cycle permanently, a phenomenon referred to as senescence, or triggered apoptosis. The mechanism of action of chemotherapeutic agents and the dose and type of RT determines the spectrum of DNA damage induced by treatment. As discussed earlier, complex DNA lesions are the most challenging type of damage for a cell to repair. This section focuses on whether there is evidence linking efficacy of chemotherapeutic drugs or RT to the type of DNA damage they incur. Additionally, a discussion is made on evidence from literature that highlights the drawback of using these agents for therapy, since normal cells affected by these insults to their DNA can also lead to a second primary cancer development.

The therapeutic index is high when molecular targets overexpressed specifically in tumor cells can be targeted by small molecule inhibitors. Multi-kinase inhibitors have dramatically improved patient survival in hematologic malignancies, while drugs targeting cancer specific mutations have improved survival in select patient populations. In the mid-1970s, 5 year survival estimates were at 41% for patients diagnosed with acute lymphocytic leukemia while they are reported at 71% for patients diagnosed between 2006 and 2012. Similar improvement has been witnessed for chronic myeloid leukemia, 22% to 66% in the same time intervals [111]. However, currently available standard chemotherapeutic agents and even the latest technologies in radiation physics fail to qualify as curative options for several cancer types. Commonly used chemotherapy regimens include platinum based DNA alkylating agents, topoisomerase poisons, antimetabolites, microtubule inhibitors, antitumor antibiotics, proteasome inhibitors etc. [112]. Antitumor antibiotics include a class of drugs called anthracyclines that inhibit pathways that generate DNA nucleotides. Non anthracycline drugs in this class include a compound called bleomycin. Bleomycin portrays the strongest evidence for clustered DNA damage being used as the mechanism of action for a chemotherapeutic agent. The mechanism of action of bleomycin and the similarities in base damage produced when compared with IR makes it a “radiomimetic” chemotherapeutic [113]. The drug creates reactive aldehyde groups at the sugar moiety that is capable of reacting with cytosine residues in its proximity and creating clustered DNA damage. Use of bleomycin has been inhibited due to severe pulmonary toxicity [114] and risk of pulmonary fibrosis despite tolerable myelotoxicity. Since the clinical trials establishing the correlation between bleomycin use and pulmonary toxicity in the 1980s [115,116] there has been years of research that indicates the importance of ROS at the site of action in propagation of the oxidative DNA damage. Even low levels of ROS have been reported to cause GI via NHEJ-mediated DNA repair [117].

One of the major treatment modalities for several types of cancers is RT, and ROS and clustered DNA damage are thought to be critical to mediate the effect of IR. Many decades of experimental research in cellular and molecular radiation biology provide evidence suggesting that nuclear DNA is the critical target of IR, and both the initial and residual levels of DSBs are linked to the biological effects of radiation, and that DNA damage and repair is relevant to carcinogenesis [118]. Precise delivery of radiation beams to site of solid tumors has improved with advances in medical physics and engineering. Among these, the use of proton beams as an alternative to traditional high energy electrons has at least in theory, improved accuracy of targeting and reduction in surrounding tissue toxicity. Long term follow-up data for significant patient cohort sizes will enable us to compare the potential benefits of proton beam therapy. OCDLs are a hallmark of IR although their endogenous levels are relatively low [119,120]. Radiation dose and quality dictates the complexity of DNA damage induced by the particle. Increasing dosage and LET (linear energy transfer) correlates with higher accumulation of clustered lesions in cancer cells [25,64]. The recruitment kinetics of DNA repair proteins is dependent on the level of LET [121]. The fact that DNA repair capability is compromised with increasing complexity of damage underscores the importance of these lesions in therapy [24,122]. One of the significant and therapeutic advantages of high LET IR is that there are extensive amounts of clustered damage leading to increased relative biological effectiveness (RBE) vs. both photon-based and even proton-based modalities [123]. As recently reviewed by Mohamad et al. [124], comparison of conventional photon-based external beam radiation to carbon ion radiotherapy reveals that carbon ions result in a better and more targeted-to-the-tumor dose distribution, higher LET and RBE. This improved RBE relates to the unique high-LET radiation-induced complex DNA damage that overpowers the DNA repair system of tumor cells as also showed for example by earlier studies using human monocytes exposed to ^56^Fe ions (LET=148 keV/µm) [125]. The use of carbon or other high-LET particles maybe a solution in the case of difficult to treat tumors, including those that are hypoxic, radio-resistant, or located deeper in the body [124]. On the history of carbon-ion based RT, one of the pioneers was the National Institute of Radiological Sciences (NIRS) which started treating patients with beams in the Heavy Ion Medical Accelerator (HIMAC) in Chiba, Japan in 1994. Following Japan, Germany in 1997 in the Gesellschaftfür Schwerionenforschung (GSI) in Darmstadt, treated their first patient and later in the Heidelberg Ion Therapy Center (HIT) in 2009. Therefore based on clinical evidence, mostly originating from Japan and Germany, high-LET radiations maybe a promising RT modality with limited radiation toxicity [124].

The precise contribution to the effects of clustered DNA lesions after proton treatment on cells is a matter of debate that remains to be studied in further detail [126,127,128]. The highest LET along the path of a proton beam around the Bragg peak has been reported to correlate with maximum complexity of DNA damage [129].

The rise in the population of cancer survivors has led to a better understanding of the effects of RT to treat cancer patients [130]. A significant portion of cancer survivors are patients with a history of childhood cancer. According to the American Cancer Society (ACS) the 5-year survival rate for childhood cancer patients is now over 80%. However, exposure to radiation treatment can lead to the occurrence of secondary primary cancers (SPCs) in the future. An analysis of thyroid cancer in childhood cancer survivors showed that the relative risk of thyroid cancer in these patients increased linearly with the dose of radiation for treatment through 10 Gy [131]. The relative risk of thyroid cancer post RT was lowered at high treatment doses. Another study looking at chest RT to treat childhood cancers showed an increased risk of breast cancer in these patients [132]. Especially treatment involving whole-lung irradiation increased this risk. This shows the importance of localized RT to reduce the risk of SPCs. The need for improvement in RT techniques that spare normal tissue is also highlighted by the incidence of metachronous cancers (multiple primary cancers developing at intervals) in adults. The incidence of secondary primary lung tumors increased by a stark 8.5% per Gy in women who had undergone RT for breast cancer [133]. An analysis of prostate cancer patients also told a similar story. Patients with prostate cancer undergoing RT had an increased overall risk of developing hematologic, liver, esophageal, and urinary bladder cancers [134]. Certainly, the induction of DNA damage by RT is an important factor towards the prediction of SPCs. Recent studies for example show that simulated radiation-induced persistent telomere-associated DNA damage foci can be used to predict excess relative risk of developing secondary leukemia after fractionated radiotherapy [135]. In general, the incidence of secondary malignant neoplasms (SMN) depends on several factors like patient’s lifestyle, genetic susceptibility, DNA repair efficiency and radiosensitivity of the patient or specific organ [136].

The advent of proton beam therapy (PBT) brings new promise of reduced radiation treatment related morbidity by minimizing the dose to critical normal tissues [137]. Proton therapy has shown great therapeutic potential in treating various adult malignancies including the central nervous system and gastrointestinal tract, but with uncertain benefits for example for lung cancers. At the same time, it has been estimated that excess fatal SPCs may be further reduced with proton therapy by two-thirds compared to conventional photon therapy [138]. However, evidence that PBT reduces occurrence of metachronous cancers is limited. A study looking at PBT for advanced cholangiocarcinomas showed gastrointestinal toxicities and early metastatic progression still remains a treatment obstacle [139]. Another study looking at cardiac events post RT in patients with thymic malignancies, showed that the lower dose to organs with PBT reduced the occurrence of major cardiac events post treatment [140]. A long-term follow-up of patients with pediatric tumors showed fewer late adverse events and a reduced risk of metachronous malignancies with PBT [141]. Similar studies seem to indicate that the reduced dose to normal structures with PBT as opposed to intensity-modulated RT is better tolerated by the patient population [142]. But, it may be too soon to draw a conclusion on the benefits of PBT over traditional photon RT. A study comparing RT usage trends in men with localized prostate cancers pointed differences in demographic and prognostic factors between patients treated with proton and photon RT [143]. Thus, although the physical theory of it may indicate to clear benefits, there is a need for more long-term assessments and more studies in general looking at the potential profits of PBT over traditional RT.

## 5. Concluding Remarks

In this mini-review, we present the idea of complex (clustered DNA damage), the signature of IR, by a different perspective that of its clinical implications and its involvement in the route to carcinogenesis. As recently discussed in Pateras et al. [109], enthralling evidence supports the idea that DNA damage response and repair (DDR/R) and immune response signaling networks work together towards the proper function of organisms and homeostasis. We believe that there is a strong linkage between the induction of complex DNA damage, deficient or incomplete DNA repair, constant DDR/R triggering and the continuous activation of the immune system. This vicious relationship which is usually accompanied by GI can be considered without any doubt as the major pathway leading to carcinogenesis [144,145]. Chronic inflammation which is synonymous to the activation of innate immune system can lead to the downregulation of DNA repair pathways and cell cycle checkpoints due to the release of inflammatory mediators and ROS which can lead to GI [146]. Towards this direction, Colotta et al. suggested a few years ago, that cancer-related inflammation can promote GI by the various inflammatory mediators, leading to accumulation of random genetic modification in cancer or healthy cells. According to the authors, this cancer-relating inflammation represents the seventh hallmark of cancer [147] in addition to the six hallmarks suggested initially by Hanahan and Weinberg [148]. The understanding of the mechanisms that repair-resistant DNA damage is processed by the cells will benefit significantly therapeutic applications maximizing tumor killing and minimizing radiation toxicity for the cancer patient under RT.

Therefore, one can easily understand the importance of detecting correctly not only DSBs but also all other forms of non-DSB clustered lesions (OCDLs) and especially in the context of chromatin. A special effort must be made by the scientific community to optimize the specificity and accuracy of all current methodologies for the detection of complex DNA damage in situ and even better under live-cell imaging conditions.

## Abbreviations

The following abbreviations are used in this manuscript:

AP	Apurinic/apyrimidinic
BER	Base excision repair
bp	Base pairs
DAMPs	Damage-associated molecular patterns
DNA	Deoxyribonucleic acid
DDR/R	DNA damage response and repair
DSB	Double strand break
GI	Genomic instability
HMGB-1	High-mobility group box 1
IL	Interleukin
IR	Ionizing radiation
HR	Homologous recombination
LET	Linear energy transfer
MC	Monte Carlo
NHEJ	Non-homologous end joining
NTE	Non-targeted effects
OCDL	Oxidatively-clustered DNA lesions
PBT	Proton beam therapy
PRPs	Pattern recognition receptor
RNS	Reactive nitrogen species
ROS	Reactive oxygen species
RT	Radiotherapy
SPCs	Secondary primary cancers
SSB	Single strand break
TLRs	Toll-like receptors
